# Large Angle Congenital Esotropia in a Child With Kabuki Syndrome: A Case Report

**DOI:** 10.7759/cureus.8445

**Published:** 2020-06-04

**Authors:** Talaat Hamdi, Ahmed Basheikh

**Affiliations:** 1 Ophthalmology, Jeddah University, Jeddah, SAU; 2 Ophthalmology, King Abdulaziz University, Jeddah, SAU

**Keywords:** large congenital esotropia, kabuki syndrome, pediatrics

## Abstract

Kabuki syndrome is an uncommon genetic disease associated with skeletal, cardiac, neurological, and ocular manifestations. Strabismus is an ophthalmic manifestation of Kabuki syndrome; however, it is infrequently documented in detail. We report a case of Kabuki syndrome in a patient who presented with large angle congenital esotropia. This case report highlights the importance of early eye examinations and subsequent interventions in patients diagnosed with Kabuki syndrome.

## Introduction

Kabuki syndrome is a rare congenital genetic disease with an estimated prevalence of 1 in 32,000 Japanese births [1]. The name originates from the characteristic faces present in patients, which are similar to those of actors in traditional Japanese Kabuki theater. Other names that have been used include Niikawa-Kuroki syndrome and Kabuki make-up syndrome. This disease is believed to be caused by mutations in the KMT2D and KDM6A gene, with multiple patterns of inheritance, including both autosomal dominant and X-linked inheritance patterns [1, 2]. In Saudi Arabia, Kabuki syndrome is not well reported due in part to rarity as well as lack of disease awareness. To our knowledge, this is one of a few case reports that document a large congenital esotropia in a child with Kabuki syndrome in Saudi Arabia.

## Case presentation

A two-year-and-ten-month-old boy presented with notable developmental delay, including requiring support to sit, lack of social smile, inability to recognize voices, and inability to pass objects between hands. The child was born at 39 weeks gestational age through planned cesarean section. The patient first presented to the hospital at three months of age with a weight of 4.3 kg, height of 50 cm, and head circumference of 40 cm. At the time of the present evaluation, his height was 89 cm (2nd percentile, UK-WHO growth chart) and weight was 10 kg (less than the 0.4th percentile), indicating severe growth retardation.

Perinatal history was unremarkable, and the family history was negative with no history of consanguinity. The patient was admitted to the hospital twice due to atypical febrile convulsions, was diagnosed with hypothyroidism, and prescribed thyroxine. The patient presented with dysmorphic features including down seated posture, prominent ears, hyperpigmented areas of the skin, high arched palate, wide space between the left metatarsal bones, and downturned mouth. Echocardiography showed a small restrictive ventricular septal defect (VSD) that was asymptomatic. Genetic team consultation was performed, which confirmed the diagnosis of Kabuki syndrome.

**Figure 1:**
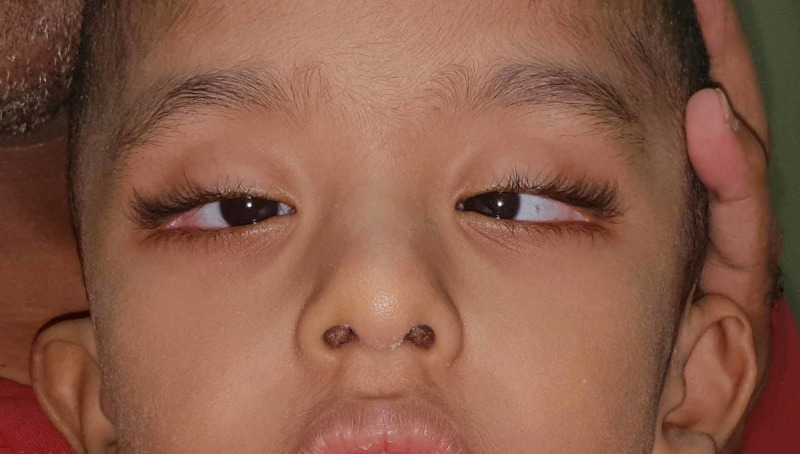
Large esotropia in Kabuki syndrome patient. Photograph of the patient in this case demonstrates the characteristic facial features associated with Kabuki syndrome, including long palpebral fissure, eversion of the lateral one-third of the lower eyelid, arched eyebrows with sparseness in the lateral one-third, low set ears and depressed nasal tip. In addition, a large angle esotropia that has been present since birth is shown.

Ophthalmologic examination conducted by a pediatric ophthalmologist at around eight months of age showed a large congenital esotropia (ET) with a variable angle, and the Krimsky test showed about 70 prism diopters (PD) at near in the primary position. The patient's visual acuity showed a mild grimace to light about each eye separately, with no fixation, and no following of objects. Retinal examination showed no aberrant abnormality besides mild disc pallor. The pupils were reactive with no afferent pupillary defect. Atropine refraction at eight months showed OD: +0.75 D sphere +0.75 D cylinder X 100, OS: +0.25 D sphere + 0.50 D cylinder X 85. Repeat of atropine refraction at two years of age showed OD: -4.50 D sphere +1.25 D cylinder X 75, OS: -3.75 D sphere +0.75 D cylinder X 115 with myopic astigmatism that was corrected with glasses. Vision at the time of presentation was central, steady, and maintained in both eyes; the extraocular muscle ductions and versions showed minimal abduction defects in both eyes. The Krimsky test with correction showed around 45-50 PD at near. Anterior segment and retinal examination remain within normal limits at two years of age. Other ocular findings seen in the patient were long palpebral fissures, sparse arched eyebrows, eversion of the outer third of the lower eyelids, epicanthal folds, and bilateral ptosis as shown in Figure 1.

## Discussion

Kabuki syndrome presents with a wide variability in clinical signs. Nonetheless, based on the analysis of 62 patients by Niikawa et al., the diagnosis of Kabuki syndrome requires five fundamental features: 1) characteristic face (100%) with long palpebral fissures, eversion of the lateral one-third of the lower eyelid, arched eyebrows with sparseness in the lateral one-third, prominent large ears and depressed nasal tip; 2) skeletal abnormality (92%); 3) dermatoglyphic abnormality (93%); 4) mental retardation (92%), and 5) short stature [3]. These features were all observed in this patient and led to a diagnosis of Kabuki syndrome.

Kabuki syndrome is associated with many ocular findings with prevalence ranges of 38% to 61% [4]. These include but are not limited to sparse hair in the lateral part of the eyebrows, strabismus, ptosis, amblyopia, refractive errors, sixth cranial nerve palsy, blue sclera, cataracts, retinal pigmentary changes, coloboma of the iris and retina, congenital corneal staphyloma, and Peter’s anomaly [2, 4-7].

A previous expansive literature review found that strabismus was present in about 22% of 300 patients with a confirmed diagnosis of Kabuki syndrome [8]. However, these cases lack detailed documentation and only three cases reported the degrees of deviation most of which were found to be at small angles (12-25 PD). Additionally, these cases described older age patients, ranging from five to nine years of age [9]. Sharma and Dave reported one case of esotropia subsequently treated with 4-mm bimedial rectus recession with an approximate angle of deviation of 25 PD [9]. We report a case with a larger angel strabismus at a young age at approximately 70 PD, which decreased over time with full refractive error correction to approximately 45-50 PD.

## Conclusions

In conclusion, we advocate the need for careful monitoring of the case before intervention as the strabismus angle might change over time from the first presentation. Besides, we emphasise the important need for an early eye examination in patients diagnosed with Kabuki syndrome to prevent visual impairment.
